# A psychoacoustic application for the adjustment of electrical hearing thresholds in cochlear implant patients

**DOI:** 10.1371/journal.pone.0223625

**Published:** 2019-10-11

**Authors:** Johannes Plesch, Benjamin P. Ernst, Sebastian Strieth, Tobias Rader

**Affiliations:** 1 Division of Audiological Acoustics, Department of Otolaryngology, University Medical Center, Mainz, Germany; 2 Department of Otolaryngology—Head and Neck Surgery, University Medical Center, Mainz, Germany; Medical University Hannover; Cluster of Excellence Hearing4all, GERMANY

## Abstract

**Objective:**

Fitting cochlear implants, especially the precise determination of electrical hearing thresholds, is a time-consuming and complex task for patients as well as audiologists. Aim of the study was to develop a method that enables cochlear implant (CI) patients to determine their electrical hearing thresholds precisely and independently. Applicability and impact of this method on speech perception in noise at soft speech levels were evaluated.

**Method:**

An adaptive psychoacoustic procedure for precise hearing threshold determination (precT) was implemented using MatLab (MathWorks) and a graphical user interface was created. Sound signals were calibrated with a CIC4-Implant-Decoder. *Study design*: A prospective study including 15 experienced adult cochlear implant users was conducted. Electrical hearing thresholds were determined using the automated precT procedure (auto-precT). Speech perception in noise at 50 dB SPL presentation levels was measured for three conditions: (P1) T-levels kept at the previously established T-levels; (P2) T-levels set to the hearing thresholds determined using auto-precT application; (P3) T-levels set 10 cu below the values determined with auto-precT.

**Results:**

All subjects were able to perform the auto-precT application independently. T-levels were altered on average by an absolute value of 10.5 cu using auto-precT. Median speech reception thresholds were significantly improved from 2.5 dB SNR (P1) to 1.6 dB SNR (P2, p = 0.02). Speech perception was lowest using the globally lowered T-levels, median 2.9 dB SNR (P3, not significant compared to P1 and P2).

**Conclusion:**

The applicability of the developed auto-precT application was confirmed in the present clinical study. Patients benefited from adjusting previously established T-levels to the threshold levels determined by the auto-precT application. The integration of the application in the clinical fitting routine as well as a remote fitting software approach is recommended. Furthermore, future possibilities of auto-precT include the implementation of the application on tablets or smart phones.

## Introduction

### Overview of the research topic

A precise fitting of cochlear implants is essential for maximum speech perception. Two important fitting parameters are the threshold and the comfortable levels, T- and C-levels respectively. The T-levels are usually set to the lowest electrical current that evokes a hearing sensation and the C-levels are set to a current that evokes sounds with a comfortable loudness. Interestingly, Vaerenberg et al. [1] found in a global survey that it is common practice to measure T- and C-levels only for a few electrodes and then interpolate these values for the remaining electrodes. This reflects well-known obstacles in everyday clinical routine. C-Levels need to be well-adjusted, so that all acoustic information of speech is received well. This enables the patient to perceive all frequencies equally loud without evoking excessive loud hearing sensations. Accurate T-levels are required in order to perceive soft sounds. The importance of the ability to perceive sounds for the comprehension of soft speech has been shown in numerous studies [2–4]. It was also stated by several authors that the adjustment of T-levels for individual electrodes is beneficial [5–7]. The adjustment of precise C- and T-levels still poses a problem in cochlear implant fitting. For the initial C-levels setting, it is common clinical practice that the audiologist increases the stimulation intensity, starting at zero, until a comfortable loudness is achieved. Subsequently, the loudness is compared with the adjacent electrodes and adjusted as needed.

For several reasons the accurate determination of the electrical hearing threshold is a demanding task for audiologists as well as patients. During the first months after implant activation, patients need to get used to the new kind of hearing with the CI. This makes the challenging task of determining hearing threshold even more difficult. When audiologists present stimuli close to hearing thresholds, it is often hard for patients to judge whether they actually heard a tone or whether it was just a ‘phantom sound’. Furthermore, CI patients frequently suffer from tinnitus which makes it very difficult to differentiate which sounds are endogenous or exogenous.

Many different concepts of determining the electrical hearing threshold have been discussed (e.g. Skinner et al. [8]; van Wieringen and Wouters [9]) and there has not been an agreement on a gold standard so far. In a conference paper, Mewes and Hey [7] mentioned the widely used clinical routine of behaviorally measuring hearing thresholds, which is also used in our clinic. Initially the stimulus level is lowered from a clearly detectable level to the point with no perception. Next, the stimulus is increased until a sound is perceived again. To confirm the determined hearing threshold, the stimulus level is lowered once more by a smaller step size until no hearing sensation is perceived and then increased again until the sound is detected, respectively.

With the intention to free audiologists from the time-demanding task of behaviorally measuring hearing thresholds for all electrodes (implants by COCHLEAR have 22 electrodes), so-called streamlined fitting procedures have been developed ([6, 10]). In order to streamline the CI-programming some audiologists use the electrically evoked whole nerve action potentials (ECAP) as a parameter. Alternatively, hearing thresholds are behaviorally measured only for some electrodes and interpolated for the rest.

Recently Rader et al. [11] presented an innovative adaptive method for determining precise electrical hearing thresholds (precT) and evaluated the impact of the precise fitting on speech perception at soft levels. The electrical hearing thresholds were determined by applying an alternative forced choice (afc) method using the established fitting software Custom Sound (COCHLEAR, Macquarie, Australia). The results of this approach were very promising, as the concept led to a significant improvement in the perception of soft speech.

The objectives of this research project were derived from the benefit of precisely determining hearing thresholds as reported earlier (Rader et al. [11]) and the demand for improvements in clinical workflow routines. Consequently, following steps were taken:

1. The precT procedure was automated and implemented into a MatLab program. Thus the ‘auto-precT’ procedure can be performed, independently of the clinical fitting software and without an audiologist, just by patients themselves.
2. The new method was evaluated in a clinical study regarding feasibility and speech recognition outcome.

## Materials and methods

### Implementation the auto-precT method

#### A two stimuli approach for precise threshold determination

**Fig 1:**
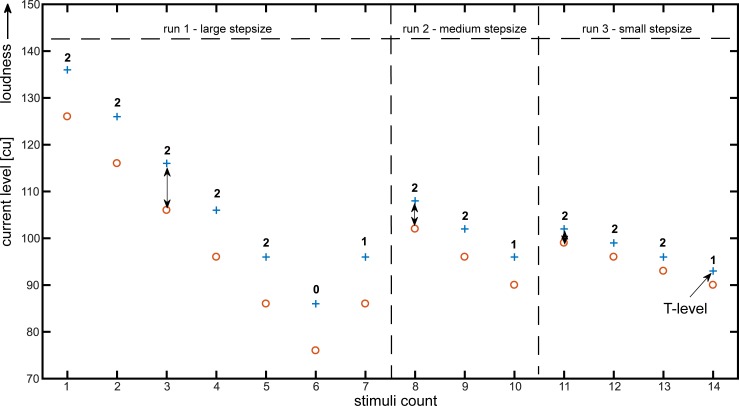
Exemplary iteration of the precT procedure for one electrode. The bold numbers indicate the count of perceived sounds. The double-headed arrows indicate the applied step size. The precT method was derived from earlier reports (Rader et al. [11]).

The auto-precT application determines the threshold levels with an iterative adaptive three alternative choice method. T- and C-levels are measured in current units (cu), the unit for current levels. The relationship in between current unit and microampere is logarithmic, so that a change in current levels can be linearly transformed to a current change on the dB scale. The newly developed software, based on the precT method as proposed earlier (Rader et al. [11]), repeatedly presents two stimuli with the same frequency, but with different current levels. After the presentation of the two stimuli subjects were asked how many sounds they heard. Given the answer is ‘two’, it can be assumed that both stimuli were above the hearing threshold and the stimuli levels are subsequently decreased by the step size set before. If no stimulus was perceived, the stimuli levels are increased by the chosen step size. Given the answer is ‘one’, the hearing threshold is presumably in between the two stimuli. In this case, the first algorithmic circle is over and a second run starts. For each electrode there are three repetitions with sequentially smaller step sizes in between the current levels of the stimuli. ln First, the step size is set at 10 current units (cu), then at 6 cu and finally at 3 cu. When the patient perceived only one stimulus at a time the run is finished. In this case, the step size in between the stimuli is decreased. Subsequently, the next run starts at the recent level plus two times the step size. The last stimulus that was heard in the third run is saved as the hearing threshold and is later set as the T-level for that electrode. The patient works through this procedure for every electrode in a pseudo-randomized order. Fig 1 shows an exemplary run for one electrode.

#### Hardware setup and software settings

**Fig 2:**
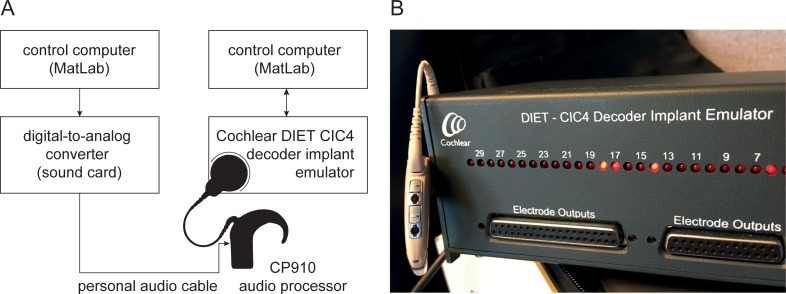
Hardware setup for audio processor and audio signal calibration. (A) Scheme of the setup. (B) Cochlear DIET—CIC4 decoder implant emulator with attached sound processor CP910. The DIET visualizes which electrodes are being stimulated and logs the stimulation data.

One aim of the study was to develop a software and a hardware setup that allows running the proposed precT procedure without the clinical fitting software. Thus, it needed to be ensured that the newly developed application can evoke specific current levels at specific electrodes. Therefore, in order to calibrate the setup, the following method was developed: A control computer with MatLab was connected to a sound card in which a so-called personal audio cable, PAC (COCHLEAR) in the following, was plugged in. This connected the sound card with a CP910 audio processor (COCHLEAR). In order to measure the current evoked by an audio signal, generated with MatLab, a Decoder Implant Emulator (DIET, COCHLEAR, Macquarie, Australia) was used. The DIET can be connected to an audio processor and measure the stimulation data. Fig 2 displays the whole hardware setup for the calibration. All measurements were performed in a soundproof room. The audio signal was converted with a high-quality 24-bit, 8-channel AD-DA converter (RME Fireface UC, Haimhausen, Germany) and then transmitted to the Cochlear CP910 audio processor via the PAC. The audio processor was connected to the DIET and linked to a second control computer in order to log the stimulation data for every electrode. Using the DIET it was possible to correlate the generated stimuli with the electric current induced.

For the calibration as well as for the clinical study, a standard CP910 audio processor with a standardized ‘flat map’ was used with all T- and C-levels globally set to 82 and 166 current units. A flat map is used so that the auto-precT procedure without knowledge of the C-levels can be performed with a non-individually programmed processor. Cochlear’s ACE strategy was used as a stimulation strategy (e.g. Skinner et al. [12]).

The other settings of the flat map were as follows: The value for ‘maxima’ was set to ‘1’, in order to ensure that only one electrode was stimulated at a given time by the sinusoidal audio input via PAC. The pulse width was set to 25 μs and the stimulation rate to 900 pps. These values are based on a clinical database of fitting parameters of cochlear implants using *Cochlear Ltd*. Devices [13]. The whitepaper states that about 95 percent of implants have the threshold levels set within that range. The other map parameters remained on factory default; especially the values for the sound pressure level (SPL) limits, the threshold-SPL (T-SPL) and comfortable-SPL (C-SPL), were set at 25 dB and 65 dB. Input sounds with a SPL below the T-SPL do not lead to a stimulation and input sounds with a SPL above the C-SPL lead to a stimulation at the set C-level.

#### Calibrating electrode specific stimuli

**Fig 3:**
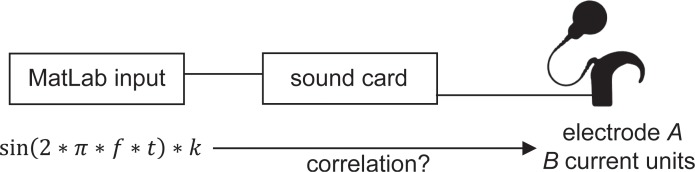
Calibration approach. Stimuli were sinus signals created in MatLab with the specific frequency *f* and an attenuation factor *F(k)*. The audio signals stimulated a specific electrode *A* with a current level of *B* current units. The stimulation data was logged with the DIET, so the correlation of the attenuation factors and the current level the audio signals evoked could be evaluated.

In order to stimulate specific electrodes, sound files with the middle frequencies of the corresponding band-pass filters were created in MatLab. As the value for ‘maxima’ on the audio processor was set to ‘1’, only the targeted electrode was stimulated. It was challenging to generate and calibrate stimuli evoking the intended specific current level, because the correlation in between the digital input in MatLab and the current level it evokes was unknown at first. In order to generate stimuli that evoke different current levels, an approach with ‘attenuation factors’ *F(k)* was taken (Fig 3). The sinusoidal stimuli were multiplied by the attenuation factors and the evoked current levels could be measured using the DIET. Thereby, the correlation between the applied attenuation factors and the current levels could be evaluated.

As a premise, each attenuation factor *F(k)* should lower the sound signal by 1 dB. Subsequently, the attenuation factors could be calculated with the following formula: *F(k) = 10*^*-k/20*^.

**Fig 4:**
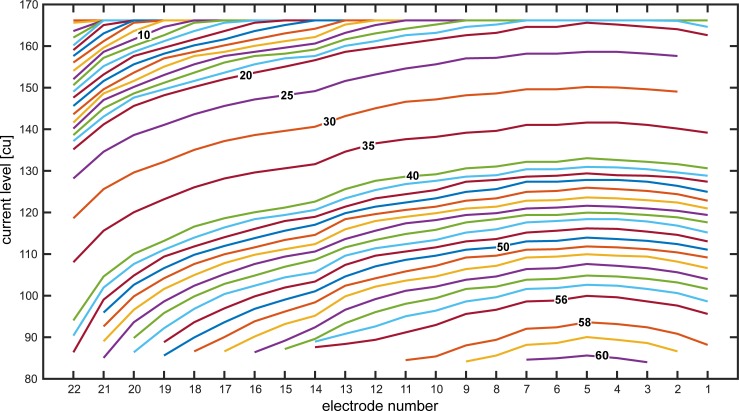
Correlation between current levels evoked by the stimuli generated with MatLab and their attenuation factors (bold numbers). Stimulation data was measured with the DIET. Between factor 20 and 40, measurements were only performed for every 5^th^ attenuation factor and for factor 57 no data was measured. The values in between were interpolated.

The sound card settings were chosen so that an unattenuated stimulus evoked current levels of 166 cu (= C-level, maximum stimulation level) at every electrode. The PAC connected to the audio input of the audio processor have a frequency-specific transfer function. The frequency range of the input signal is divided into different stimulation channels. This results in different attenuation factors for the stimulation channels. In order to evaluate the correlation between the current levels evoked by the stimuli and their attenuation factors, the stimulation data were measured with the DIET. Fig 4 displays the correlation for all electrodes. The bold numbers indicate attenuation factors. Between factor 20 and 40, measurements were only performed for every 5^th^ attenuation factor. The values in between were interpolated.

A calibration matrix was created with the measured data (Table 1). With the calibration matrix the attenuation factor that is needed to generate a stimulus that evokes a specific current level could be interpolated.

**Table 1:** Calibration matrix.

	el = 22	…	el = 1
F(1)			
…		current levels	
F(65)			

Calibration matrix with the current levels that have been measured for the different attenuation factors.

**Fig 5:**
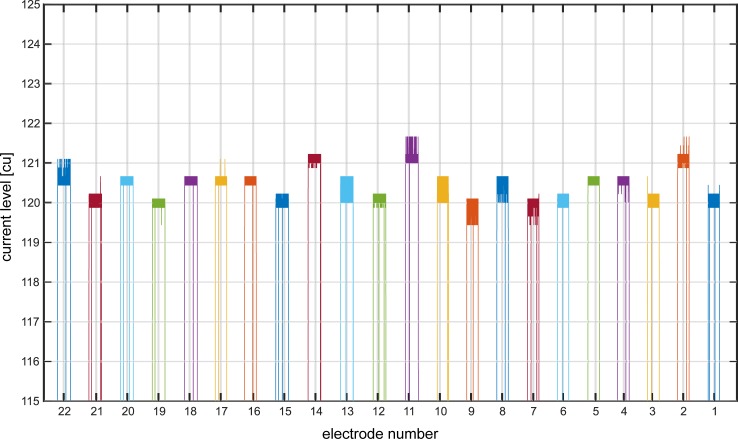
Exemplary calibration assessment. The audio signals created with a MatLab script were to evoke a current level of 120 cu. The figure shows the stimulation data measured with the DIET. The calibration is sufficiently accurate (standard deviation was 0.37 cu; that is less than 1% of the average dynamic range). The small deviations are due to rounding of the attenuation factors, minor fluctuations at the individual electrodes are caused by the transformation of the audio signal.

The accuracy of the calibration that has been previously described was assessed in the following way: the hardware set up was in a soundproof room. A MatLab script was run and generated audio signals that stimulated every single electrode, one after another, with the same intended current levels. The stimulation data was logged with the DIET. Hence, the evoked current levels could be compared with the MatLab input. The assessment showed that the calibration and the hardware setup were sufficiently accurate (Fig 5). Only small deviations (standard deviation was 0.37 cu; that is less than 1% of the average dynamic range) and minor fluctuations were observed. The reason for the deviations is the rounding of the attenuation factors. The minor fluctuations at the individual electrodes are due to the setup. When using the clinical fitting software to stimulate single electrodes, the stimuli are directly generated by the sound processor. In our setup, the stimuli were evoked by an audio signal. The processing of the audio signal causes the minor fluctuations.

After successful calibration of the setup, the precT-procedure was implemented in MatLab. Several scripts, functions and a graphical user interface (GUI) were developed.

The aim was to create a self-explaining application, so a GUI that is easy to understand and interact with was developed. When the auto-precT procedure starts, the patient’s ID is entered at first. Before the patient begins with the afc procedure, a window with a text explaining the program is displayed. Next, an example is shown in which two stimuli are presented–one at the starting current level and one 10 cu below. After that, a window appears asking whether the patient has any further questions and if he/she is ready to start. Then the procedure starts. A window with an orange box appears. Only during the time the orange box is visible stimuli can be perceived. To prevent a habituation effect the order of the stimuli was randomized, so at times the stimulus with the higher current level is played first and at times the stimulus with the lower current level. Furthermore, the time before the first stimulus is presented is randomly varied (1 to 2 seconds) and as well the time between the first and the second stimulus (1.4 to 2.4 seconds). Also the order of the electrodes was pseudo-randomized with two lists. After the presentation of the stimuli, a response window is shown, asking how many sounds have been heard. The patient presses either zero, one or two and the next iteration starts. After the patient has finished the three runs for an electrode, a window appears with a button saying, ‘continue with the next electrode’. This was implemented to give the patient an opportunity to take a break if needed, as the procedure requires a lot of concentration.

### Evaluation of the auto-precT application

After the implementation of the auto-precT application a clinical study was conducted. The study was approved by the local ethical review board (Landesärztekammer Rheinland/Pfalz, 837.462.17(11296)) and all participants gave their written informed consent before the start of any study-specific procedure.

The study consisted of three parts. First, the speech perception of the subjects with their current sound processor settings was tested (P1). Then the subjects followed the newly developed application and determined their threshold values on their own. After that a map with T-levels set to the determined hearing thresholds (P2) and another map with T-levels 10 cu lower than thresholds determined with auto-precT (P3) were created. Subsequently, the speech perception with those maps was tested.

#### Subjects

Fifteen experienced CI-users with a CI usage from 7 to 124 months (median: 21) participated in this prospective study. The age ranged from 20 to 71 with a median of 56 years. Criterion for inclusion in the study was good speech perception with a Freiburg Monosyllable Score (FMS) of 60 percent or higher at 65 dB SPL free field presentation level. Five participants were bilaterally implanted. In this case only the better performing ear (higher scores in speech perception) was tested. The contralateral ear of subjects with residual hearing was masked with an earplug during all tests. All subjects had an implant by COCHLEAR (Macquarie, Australia) and were using a CP810, CP910, CP950 or CP1000 sound processor and the speech coding strategy ‘ACE’ [14]. The other established processor settings were following: stimulation rate 900 pps; stimulation mode MP1+2; maxima 8; pulse width 25 μs (n = 3), 37 μs (n = 11), 50 μs (n = 1). The subject demographics are shown in Table 2.

**Table 2:** Demographical data of study participants.

Subject ID	Age	Sex	Tested side	Implant use (months)	CI processor type	Implant type	Subject etiology	FMS (65dB) with CI
1	56	M	left*	14	CP 910	CI 522	Progressive	70
2	63	M	left	48	CP 910	CI 422	Progressive	70
3	51	F	left	13	CP 950	CI 522	Progressive	70
4	51	F	right*	7	CP 1000	CI 522	Progressive	100
5	62	F	Left	38	CP 810	CI 422	Infectious	60
6	53	F	Left	12	CP 950	CI 532	M. Meniere	60
7	58	M	right*	54	CP 910	CI 522	Progressive	70
8	61	F	Right	21	CP 910	CI 522	Infectious	100
9	37	M	Left	33	CP 910	CI 522	Hereditary	80
10	69	M	left*	31	CP 910	CI 512	Hereditary	90
11	56	F	left	14	CP 950	CI 522	Ototoxic	85
12	41	M	right	124	CP 910	CI24RE	Infectious	95
13	71	M	right	18	CP 950	CI 522	Progressive	80
14	30	M	right*	18	CP 910	CI 522	Hereditary	70
15	20	F	right	65	CP 810	CI 422	Ototoxic	100

FMS values of the ear, that was tested in the study

* bilaterally implanted

#### Experimental setup

For the assessment of speech perception sound presentation was conducted using a computer, equipped with a high-quality 24-bit, 8-channel AD-DA converter (RME Fireface UC, Haimhausen, Germany) connected to an active loudspeaker (KS Digital C5, Saarbrücken, Germany) placed in front of the subject. The speaker was placed in a soundproof room at 0° azimuths and a distance of 100 cm to the subjects’ ears. Free field stimuli were calibrated at listening position according to the manufacturer’s instructions using an Audio XL2 sound pressure level meter (NTI, Schaan, Liechtenstein).

#### Speech perception in noise

In order to evaluate the speech perception in noise, the closed-set German matrix test ‘Oldenburger Satztest’ (OLSA) was used. The test was conducted with a set speech level of 50 dB SPL presentation level. The noise level is adjusted after each trial according to the amount of correctly recognized words. The adaptive procedure determines the signal to noise ratio (SNR) at which 50 percent of the words were understood.

The speech perception test was conducted with three different conditions that were programmed on the study sound processor:

- (P1), T_established_: The established map that the patient was currently using. T-levels and other parameters were not changed. The T-levels had been adjusted by the common clinical procedure
- (P2), T_auto-precT_: A map with the T-levels set to the threshold values determined by the proposed auto-precT application
- (P3), T_auto-precT-10:_ A map with T-levels 10 cu lower than the determined threshold levels to simulate underestimated T-levels

Before using the auto-precT application, the subjects performed two OLSA test runs with their familiar sound processor settings (P1)–the first one was a training run in order to get used to the speech test, the second run was used for the evaluation (test 1). After the determination of the hearing thresholds using the auto-precT application, the participants carried out the OLSA again (tests 2 and 3) with the newly created speech processor settings (P2, P3). With each setting the OLSA was conducted twice. The better test result out of two was used for further analysis. Tests 2 and 3 were executed in a randomized manner with regard to the processor setting (P2, P3).

#### Hearing threshold determination with the auto-precT application

For the autonomous hearing threshold determination, the participants used the study standard sound processor that was calibrated for the procedure. Processor settings were the same for all subjects (flat map settings described in lines 169–173). Only the pulse width was changed in two cases: (1), if the pulse width set in the subject’s established settings was different from the default value in of 25 μs in the flat map–then the pulse width in the study processor was changed to individual processor value set in the established map (for three subjects the established pulse width was 25 μs, for eleven subjects 37 μs and for one subject 50 μs); (2), if threshold levels in a subjects established map were below 82 cu or above 166 cu (T- and C-levels in the ‘flat map’). When a lower pulse width is applied, a higher current level is needed to evoke the same electric charge and vice versa. So if the subject’s T-levels were out of the range of the flat map (82/166 cu), the pulse width in the study processor was altered so that the T-levels with the altered pulse width were within the range of 82 and 166 cu (pulse width in the study processor needed to be lowered for three subjects from 37 μs to 25 μs, who had T-levels below 82 cu in their established map, and to be raised for from 25 μs to 37 μs for one subject, who had T-levels above 166 cu in his/her established map). For those four subjects for whom a pulse width different from the established processor setting was applied in the study processor, the measured threshold levels needed to be converted for the evaluation in order to be comparable to the established T-levels, due to pulse width affecting T-levels. For the conversion the T-levels determined with the study sound processor were first transformed into microampere with the formula *I*_*1*_*[*μ*A] = 17*.5*100ˆ(I_*1*_*[cu]/255)*. Subsequently, the corresponding amperage (*I*_*2*_) for the pulse width in the established map (*PW*_*2*_) was calculated with following formula: *I*_*2*_ *= Q/PW*_*2*_ *= I*_1_*PW_*1*_*/PW*_*2*_. Last, the calculated amperage was transformed back into current units.

**Fig 6:**
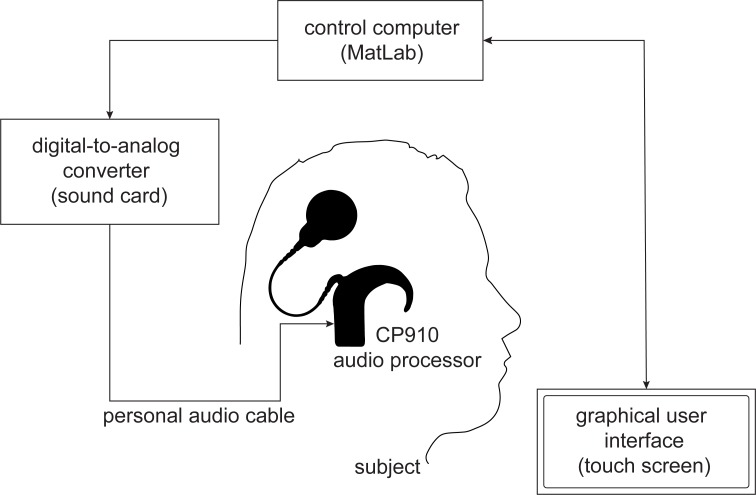
Hardware setup for the study. Stimuli generated with the MatLab program were transmitted to the audio processor with a personal audio cable. Subjects’ responses were collected with a touch screen on which the GUI of the program was shown.

The audio signals were directly transmitted from the sound card to the calibrated CP910 ‘standard speech processor’ via an audio cable. Feedback from the subject was collected using a touch screen monitor with a graphical user interface. Fig 6 shows the hardware setup for the study.

#### Subjective preference

After programming the three different map conditions (programs), they were varied during a five minute conversation with the subjects. Programs were varied every 30 to 60 seconds. The patients were asked which program they subjectively would prefer for everyday use.

#### Statistics

Statistical analyses were performed with the software IBM SPSS Statistics 23. For all test variables, a Shapiro-Wilk test confirmed normal distribution. Differences in between OLSA scores were tested with a t-test for paired samples. P-values below 0.05 were considered as significant. Unless stated otherwise, the analyses are based on the data for all n = 15 subjects.

## Results

### Feasibility and duration of the auto-precT application

All patients were able to perform the auto-precT application independently. 11 subjects performed the application taking only smaller breaks (< 5 minutes). The remaining four subjects took longer breaks, mostly because they needed to use the bathroom. Three of those four subjects needed an overall time of 53 to 56 minutes to complete application including the breaks. One subject, who took multiple breaks due to strong concentration problems, needed 69 minutes. Test duration was statistically evaluated only for the 11 subjects who took short breaks, in order to be able to compare the duration to our previous study (Rader et al. [11]). In that study the precT procedure was manually performed using the clinical fitting software Custom Sound and no breaks were reported. In the current study the 11 subjects performed the auto-precT application, executing three consecutive runs for all 22 electrodes, in an average time of 39 min (approximately 107 s/electrode), including the smaller breaks they took—that is 7 minutes less than in our previous study (Rader et al. [11]). The average time needed for the first run was 14:12 (min : s), for the second run (step size 6 cu) it was 11:24 and for the third run (step size 3 cu) 13:24. No strong correlation between age and time needed was observed.

### Threshold values determined with the auto-precT application

**Fig 7:**
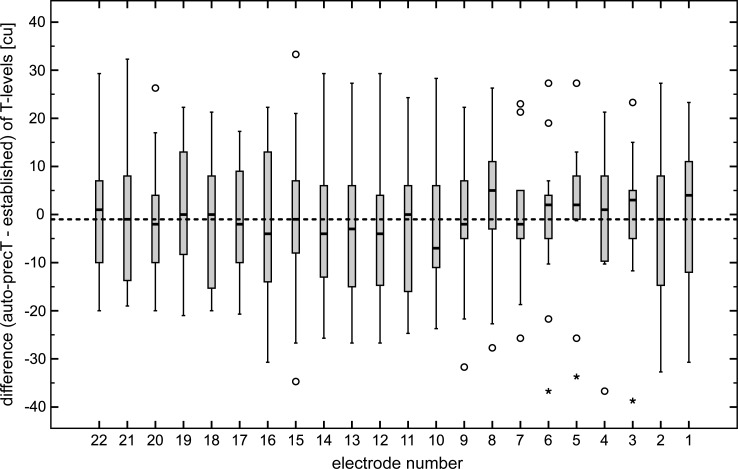
Difference in between the established T-levels and those determined with the auto-precT application. Box plot contains median, 1^st^ and 3^rd^ quartiles, minimum and maximum values. Circles indicated mild outliers (>1,5*IQR from the first or third quartile), asterisks indicated extreme outliers (>3*IQR from the first or third quartile). Dashed line indicates mean over all electrodes.

The mean difference of the threshold values determined with the auto-precT application and the established T-levels is shown in Fig 7. The mean difference is -0.7 cu, but the values are broadly spread. Threshold values determined with auto-precT were higher than the established ones for some subjects, for some they were lower. The analysis of the distribution of the mean absolute values for the difference of T-levels revealed a median of 10.5 cu, indicating that T-levels were shifted in both directions.

### Speech perception in noise at 50 dB SPL speech level

**Fig 8:**
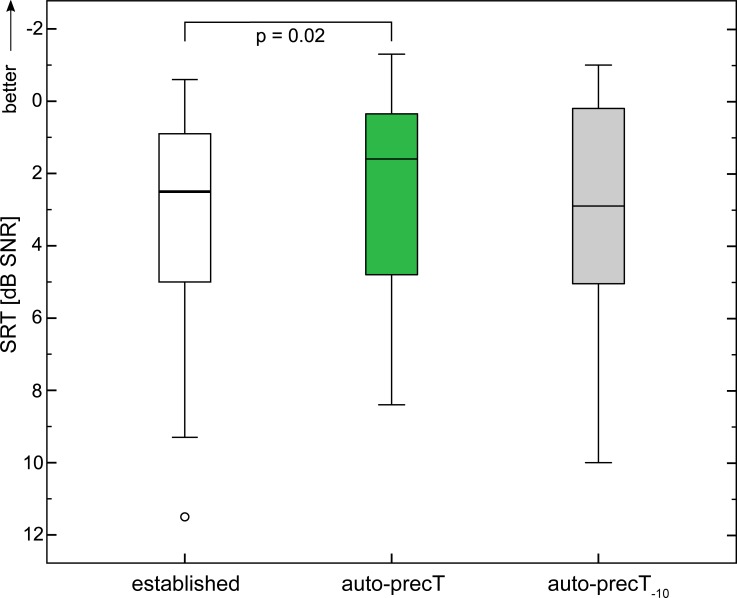
Speech reception thresholds in free field conditions (50 dB SPL speech level, noise adaptive) with three different settings for the electrical thresholds. Established: T-levels used by the subject prior to the testing; auto-precT: T-levels set to the thresholds determined with the proposed application; auto-precT_-10_: T-levels set 10 cu lower than the thresholds determined with auto-precT. Box plot contains median, 1^st^ and 3^rd^ quartiles, minimum and maximum values. Circles indicated mild outliers (>1.5*IQR from the first or third quartile).

Fig 8 displays the results of the speech perception tests in noise at 50 dB SPL speech level. Median speech reception thresholds were significantly improved (p = 0.02) with T-levels set to those determined with the auto-precT (P2) compared to the T_established_ (P1) condition from 2.5 dB SNR to 1.6 dB SNR. Speech perception was lowest with the globally lowered T-levels (P3), (median: 2.9 dB SNR).

### Subjective preference

Asked for of the preferred map conditions for everyday use, 13 of 15 subjects chose the auto-precT condition. One participant chose the established settings and one the auto-precT_-10_ condition.

## Discussion

### Impact of T-level settings on speech perception

**Fig 9:**
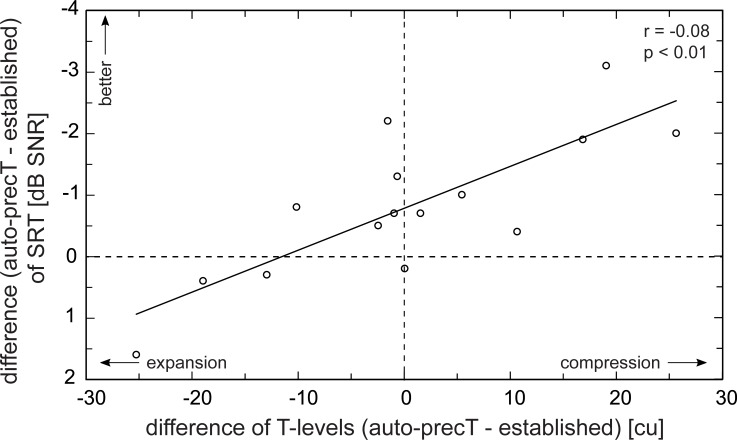
Correlation of T-levels and changes in speech perception. x-axis: difference of mean T-levels over all active electrodes. y-axis: change of speech reception thresholds measured with the established and the auto-precT settings. At first glance, compression of the electrical dynamic range (EDR) seems to lead to an improvement in speech perception at soft speech presentation levels (upper right quadrant). However, also subjects with only a small shift of the mean EDR had an improvement in speech perception, most likely as a result of the precise threshold determination for every individual electrode with the auto-precT application.

The evaluation of the impact of the T-level shifts (difference of the mean value over all active electrodes) on speech perception showed interesting results: All subjects with increased T-levels (n = 6) had an improvement in speech perception (upper right quadrant in Fig 9). Corresponding to that, most subjects with decreased T-levels had a deterioration in speech perception (lower left quadrant in Fig 9). Consequently, the results might suggest that compression of the electrical dynamic range (range in between T- and C-level, EDR) leads to an improvement in speech perception (Pearson correlation coefficient r = -0.8, p < 0.01). This observation is similar to the results of our previous study (Rader et al. [11]) where a mean elevation of T-levels by 9 cu led to an improvement in speech perception at 50 dB SPL presentation level. However, it needs to be considered that also subjects with only a small mean shift of T-levels had better scores in speech perception. This is probably due to the precise determination of threshold values for each individual electrode. T-levels were increased and decreased at different electrodes of the same subject, so there was only a small average shift. Therefore, a small average shift does not mean that only small changes of T-levels for the individual electrodes were performed. In summary, it is the precise determination of threshold levels that is beneficial for speech perception at soft speech presentation levels.

In some studies an improvement of speech perception at soft presentation levels by globally raising T-levels was described. Skinner et al. [4] reported that in their study, which they conducted with Nucleus 22 CI users, better consonant nucleus (vowel) consonant (CNC) word and sentence scores at 50 and 60 dB presentation level were observed with minimum stimulation levels raised by 2.04 dB above the clinically determined threshold values. It needs to be mentioned that the parameters (especially stimulation rate and strategy) were quite different from our study. In another publication, Holden et al. [3] reported that T-level settings above the recommendation (10% of C-Levels) of the manufacturer (Advanced Bionics CI, Auria and Harmony sound processors) may be beneficial for overall speech perception. However, in line with our findings, Holden et al. [3] concluded that an exact determination of threshold levels is necessary.

In the same study in which Botros et al. [6] introduced a (at that time) new Nucleus cochlear implant fitting suite, they presented and evaluated a new fitting methodology. They compared speech perception applying different maps. T- and C-levels were either set to the values determined with a remote fitting software (1), the nucleus fitting software (2) or to the values behaviorally measured for all electrodes (3). No significant difference in speech perception was observed. This finding seems to be in contrast to our observation, that a precise determination of threshold levels is beneficial for speech perception. However, Botros et al. [6] also stated that individual threshold measurements can improve sound quality for patients with difficulties in perceiving soft sounds. Moreover, the study showed that patients are capable of adjusting the fitting of their CI by themselves. One of the main motivations for the development of streamlined fitting methods like those used by Botros et al. [6] was to lower the effort for behaviorally measuring T- and C-levels for all individual electrodes and is to free audiologists from the time-consuming task of measuring T- and C-levels for all individual electrodes. In this regard, the proposed auto-precT application is a powerful tool. The patients can determine hearing thresholds for the individual electrodes themselves without an attending audiologist. Thus, the capacity of an audiology department is enlarged while workflow as well as the quality of the fitting is preserved or even improved. Even though the auto-precT application might be more time intensive for patients than the methods Botros et al. [6] used in remote fitting, the results of our study show that the additional time is most likely well spent.

Busby and Arora [15] investigated the impact of varying T-levels from the actual threshold levels. They tested speech perception with five different conditions: T-levels decreased by 30 and 60 percent of the dynamic range and T-levels raised by 30, 60 and 90 percent of the dynamic range, C-levels were not changed. They reported that speech perception did not significantly change with raising or lowering T-levels by 30 percent, but stated that there was ‘generally a negative impact for more compression or expansion’. Busby and Arora [15] concluded that determining threshold values precisely might not be so important. This is contrary to our findings, as in our study many subjects had an improved speech perception with the precisely determined threshold values using the auto-precT application. Actually, in our view the results from the study of Busby and Arora [15] do not allow to make a statement concerning the impact of precisely determined threshold values to be made. The T-levels they set were not threshold values measured for each electrode, but interpolated values. They determined threshold values only for six electrodes, using the Hughson-Westlake procedure (Carhart & Jerger 1959). In this procedure, after an initial descent from a clearly detectable level below the hearing threshold, the stimulus level is increased by the set ascending step size until a sound is perceived. Next, the T-level is lowered by the set descending step size until no hearing sensation is perceived anymore and then increased again until the sound is detected. This cycle is done until the level of the sound detection was the same for at least half of the iterations. Busby and Arora [15] performed the procedure with an ascending step size of 2 cu and a descending step size of 4 cu for six electrodes and interpolated the values for the remaining ones. Our findings suggest that the precise determination of threshold values for every single electrode is beneficial for speech perception, especially at low speech levels.

At the Annual Meeting of the German Association of Audiologists (DGA), Mewes and Hey [7] recently presented a study that also dealt with the impact of T-level settings on speech perception. They stated that their clinical experience contradicts the findings from Botros et al. [6] as well as those from Busby and Arora [15]. Mewes and Hey [7] conducted speech perception tests with four different conditions: T-levels set 40 cu below C-level (T = C– 40 cu), T-levels set to the hearing thresholds determined with the common clinical procedure using the Nucleus fitting software (T = HT), T-levels lowered by 25 percent of the dynamic range (T = HT– 25% DR) and T-levels lowered by 50 percent of the dynamic range (T = HT– 50% DR). They tested speech perception in quiet with the ‘Freiburger monosyllable test’ (FMS) at 70 dB and with the ‘Freiburger multi-syllable test’. In the latter, the 50% threshold of understanding was determined. Furthermore, speech perception in noise at 65 dB presentation level was assessed with the ‘Oldenburger sentence test’ (OLSA), which was also used in our study. They reported that the impact of the T-level settings on speech perception in quiet and on speech perception in noise was contrary. Lowering T-levels improved speech perception in noise at 65 dB presentation level, but worsened speech perception in quiet at low levels below 50 dB at the same time. The impact of expanding the dynamic range on speech perception in noise seems to be in contrast with our findings at first glance. However, it needs to be considered that the speech perception tests were done with different conditions than in our study (speech perception in noise was tested at 50 dB presentation level), so it is difficult to compare the results. Nonetheless, our observations support the conclusion from Mewes and Hey [7] that T-levels need to be individually optimized to reach the best possible speech perception in noise.

### Applicability

All subjects, aged from 20 to 71 years, were able to perform the MatLab based auto-precT application on a touchscreen without any problems and thereby determined their electrical hearing threshold levels completely by themselves. No clinical fitting software or help from an audiologist was needed to run the program. The subjects’ feedback to the program was entirely positive–they stated that even though the task of threshold determination requires a lot of concentration the program is ‘very intuitive’, ‘comfortable to use’ and ‘easy to understand’. The 11 subjects who took only smaller breaks (overall < 5 minutes) needed an average time of 39 minutes to run the program, 107 seconds per electrode. 125 seconds per electrode were needed on average for the manual precT program by the 18 subjects in the previous study from Rader et al. [11]. In the study of van Wieringen and Wouters [9] the time needed to determine T-levels with adaptive procedures ranged from 177 to 363 seconds per electrode. Compared to these findings, the method proposed in this study is much faster.

The auto-precT application is a good combination of both, precision and time-efficiency which are important criteria for the clinical application. Another great advantage of auto-precT is that it is self-explanatory and can be run by the patient himself. No help from an audiologist is needed while the patient is completing the program, so personnel time is saved as well. That opens up great opportunities. Consequently, the software used in this study should be refined, so that it can be run on handheld devices and smart phones.

## Conclusion

A psychoacoustic application was developed in order to allow patients to precisely and independently determine their electrical hearing thresholds, without an attending audiologist. The applicability of the program was confirmed in a clinical study. Subjects benefited from adjusting the T-levels to the threshold levels determined with auto-precT, resulting in a median improvement in speech perception in noise of -0.9 dB SNR. The auto-precT application is a useful tool for the precise determination of hearing thresholds. Thus, not only speech perception at low levels is improved with the auto-precT application, but also clinical workflow. Consequently, we recommend the integration of auto-precT in the clinical fitting as well as in remote fitting software. Furthermore, future possibilities of auto-precT include the implementation as an app on tablets or smart phones.

## Supporting information

S1 Table Table including the individual data of speech tests. (XLS)
